# Baryon decays to purely baryonic final states

**DOI:** 10.1038/s41598-018-37743-9

**Published:** 2019-02-04

**Authors:** Y. K. Hsiao, C. Q. Geng, Eduardo Rodrigues

**Affiliations:** 1 School of Physics and Information Engineering, Shanxi Normal University, Linfen, 041004 China; 2 Department of Physics, National Tsing Hua University, Hsinchu, 300 Taiwan; 3 Department of Physics, University of Cincinnati, Cincinnati, Ohio 45221 USA

## Abstract

The LHCb collaboration has presented first experimental evidence that spin-carrying matter and antimatter differ. The study looked at four-body decays of the ${{\boldsymbol{\Lambda }}}_{{\boldsymbol{b}}}^{{\bf{0}}}$ baryon. Differences in the behaviour of matter and antimatter are associated with the non-invariance of fundamental interactions under the combined charge-conjugation and parity transformations, known as *CP* violation. We discuss purely baryonic decay processes, *i*.*e*. decay processes involving only spin-carrying particles. They are yet unexplored elementary processes. Their study opens a new chapter of flavour physics in the route towards a better understanding of *CP* violation. It may help us understand the observed matter and antimatter asymmetry of the Universe.

## Introduction

The LHCb collaboration has presented first experimental evidence that spin-carrying matter and antimatter differ^1^. Differences in the behaviour of matter and antimatter are associated with the non-invariance of fundamental interactions under the combined charge-conjugation and parity transformations, known as *CP* violation. Up until then, *CP* violation had only been verified experimentally with spin-zero mesons; a brief historical review is given in ref. ^1^. As pointed out recently, the LHCb measurement marks a first step into unexplored territory^2^. It is of the utmost importance to confirm the LHCb result with higher statistical significance, analysing the larger data samples now available from the second run of the Large Hadron Collider at CERN. Furthermore, numerous other decays of beauty baryons should be studied, to establish a diverse set of observations, thereby improving our understanding of *CP* violation. Diversity of results comes in two flavours, namely from the study of a variety of different systems, and via measurements of several physical quantities sensitive to *CP* violation.

*CP* violation has far-reaching importance, being a crucial ingredient for the generation of the observed matter-antimatter asymmetry in the Universe. Unfortunately, our current theory and models can only explain a matter-antimatter asymmetry at least ten orders of magnitude smaller than the one observed. Additional sources of *CP* violation, yet to be discovered, are likely to explain the discrepancy. New sources of *CP* violation may be seen again in the quark sector or in a different sector of the theory. Since the visible Universe is made of spin-carrying particles such as the proton and the neutron, it seems natural to study purely baryonic decay processes, *i*.*e*. decay processes involving only spin-carrying particles. Any *CP* violating effects may have a more direct correspondence to the long-standing puzzle of the matter-antimatter asymmetry. These yet unexplored elementary processes may hold key information in much the same way that the study of *CP* violation with *B* mesons provided a more comprehensive understanding of *CP* violation once it got established in the decay of neutral kaons. Purely baryonic decay processes can exhibit a rich spin structure and provide complementary information to that obtained so far with mesonic decays or final states. For example, decays of baryons with spin of 1/2 or 3/2 can be used to construct time-reversal violating observables, which provide other tests of *CP* violation.

We discuss in this letter the study of purely baryonic decay processes. For each beauty baryon we present the most promising decay mode to look for, taking into account experimental constraints. Theoretical predictions are provided for some decay branching fractions and, in some cases, for the *CP* violating asymmetries.

## Results

Elementary decay processes exclusively involving baryons are only kinematically allowed with beauty baryons. These purely baryonic decays require at least three final-state particles in order to fullfil the empirical law of baryon number conservation^3^. The “lowest-ground” process is ${{\rm{\Lambda }}}_{b}^{0}\to p\bar{p}n$, discussed in ref. ^3^. We here focus our attention on the final states that are easiest to reconstruct experimentally, in full, having in mind that the LHCb collaboration is the only running experiment capable of performing the search for these processes. The lowest-ground beauty baryons of interest are the ${{\rm{\Lambda }}}_{b}^{0}$, the isospin doublet ${{\Xi }}_{b}^{0}$ and ${{\Xi }}_{b}^{-}$, and the ${{\Omega }}_{b}^{-}$. The isotriplet ${{\rm{\Sigma }}}_{b}$ baryons decay strongly, hence this family is of little interest in the study of *CP* violation in weak decay processes.

The decay ${{\rm{\Lambda }}}_{b}^{0}\to {\rm{\Lambda }}p\bar{p}$ is a fully reconstructible final state. It does involve the reconstruction of a long-lived particle, the ${\rm{\Lambda }}$ baryon. In LHCb, long-lived particles are reconstructed with lower efficiencies than single charged hadrons. Typically, an order of magnitude in selection efficiency is lost for the presence of any single fully reconstructible long-lived particle in the final state such as ${\rm{\Lambda }}$ or ${K}_{s}^{0}$, compared to the selection efficiency of reconstructing a charged hadron. Still, the final state ${\rm{\Lambda }}p\bar{p}$ seems the best way to observe for the first time a fully baryonic final state of the ${{\rm{\Lambda }}}_{b}^{0}$ baryon.

The ${{\Xi }}_{b}^{0}$ baryon can also decay to the ${\rm{\Lambda }}p\bar{p}$ final state. This decay is the most promising mode to observe a purely baryonic decay of the ${{\Xi }}_{b}^{0}$ baryon. Indeed, moving up in complexity of reconstruction, both ${{\rm{\Lambda }}}_{b}^{0}$ and ${{\Xi }}_{b}^{0}$ can decay to the ${\rm{\Lambda }}\bar{{\rm{\Lambda }}}{\rm{\Lambda }}$ final state. This final state is unique in its own right, and in particular provides a natural ground in which to study the relatively poorly known decay modes of the charmonium $c\bar{c}$ resonances to the ${\rm{\Lambda }}\bar{{\rm{\Lambda }}}$ final state. The reconstruction efficiency of three long-lived ${\rm{\Lambda }}$ baryons will unfortunately be very low, which makes the decay modes ${{\rm{\Lambda }}}_{b}^{0}\to {\rm{\Lambda }}\bar{{\rm{\Lambda }}}{\rm{\Lambda }}$ and ${{\Xi }}_{b}^{0}\to {\rm{\Lambda }}\bar{{\rm{\Lambda }}}{\rm{\Lambda }}$ out of reach until the LHCb experiment is upgraded for the years 2020s.

The search for purely baryonic decays of the ${{\Xi }}_{b}^{-}$ baryon is easiest performed looking for the mode ${{\Xi }}_{b}^{-}\to {\rm{\Lambda }}{\rm{\Lambda }}\bar{p}$. The reconstruction efficiency will be low owing to the need to reconstruct two long-lived ${\rm{\Lambda }}$ baryons.

The observation of a purely baryonic decay of the ${{\Omega }}_{b}^{-}$ will require large samples yet to be collected by an upgraded LHCb experiment. Its observation is presently out of reach. On the one hand, the production rate of ${{\Omega }}_{b}^{-}$ is rather small compared to the production of ${{\rm{\Lambda }}}_{b}^{0}$ baryons. On the other hand, the simplest decay mode is ${{\Omega }}_{b}^{-}\to {{\Xi }}^{-}p\bar{p}$, which involves the cascade ${{\Xi }}^{-}$ in the final state and hence the decay chain of two long-lived particles, as the ${{\Xi }}^{-}$ baryon is typically reconstructed in the ${\rm{\Lambda }}{\pi }^{-}$ final state. The resulting efficiency in the reconstruction of the full decay chain is very low.

### Branching fractions

As mentioned above, purely baryonic decay processes were first considered in ref. ^3^, which focused attention on the simplest decay involving the lightest possible baryons, ${{\rm{\Lambda }}}_{b}^{0}\to p\bar{p}n$. Its branching fraction is predicted to be ${\mathcal B} ({{\rm{\Lambda }}}_{b}^{0}\to p\bar{p}n)=({2.0}_{-0.2}^{+0.3})\times {10}^{-6}$^3^.

The decays ${{\rm{\Lambda }}}_{b}^{0}\to {\rm{\Lambda }}p\bar{p}$ and ${{\Xi }}_{b}^{0}\to {\rm{\Lambda }}p\bar{p}$ should be the easiest purely baryonic decay processes to observe experimentally. Their branching fractions are predicted to be $({3.2}_{-0.3}^{+0.8}\pm 0.4\pm 0.7)\times {10}^{-6}$ and $(1.4\pm 0.1\pm 0.1\pm 0.4)\times {10}^{-7}$, where the uncertainties arise from non-factorisable effects, CKM matrix elements, and hadronic form factors, respectively.

### *CP* asymmetries

The study of triple-product correlations (TPCs) in three-body decays is handicaped by the fact that the definitions of these TPCs involve the spin of one of the final-state particles. Such an issue does not happen in four-body decays, where TPCs depend only on the momenta of the final-state particles. The issue can nevertheless be overcome in specific cases, when dealing with so-called self-tagging decay modes. The decay mode ${{\rm{\Lambda }}}_{b}^{0}\to {\rm{\Lambda }}p\bar{p}$ is such a decay. The charge of the proton from the ${\rm{\Lambda }}\to p{\pi }^{-}$ decay automatically determines whether the decay is that of the ${{\rm{\Lambda }}}_{b}^{0}$ baryon or its ${\bar{{\rm{\Lambda }}}}_{b}^{0}$ antiparticle.

The direct *CP* asymmetry of the ${{\rm{\Lambda }}}_{b}^{0}\to {\rm{\Lambda }}p\bar{p}$ decay is predicted to be $(3.4\pm 0.1\pm 0.1\pm 1.0) \%$. Similarly, the direct *CP* asymmetry of the ${{\Xi }}_{b}^{0}\to {\rm{\Lambda }}p\bar{p}$ decay is predicted to be $(\,-\,13.0\pm 0.5\pm 1.5\pm 1.1) \%$. Here, the first uncertainties account for non-factorisable effects, the second reflect the experimental knowledge of the CKM matrix elements, and the third correspond to those on the hadronic form factors (see Methods for a discussion of the latter). The relatively large direct *CP* asymmetry predicted for the ${{\Xi }}_{b}^{0}\to {\rm{\Lambda }}p\bar{p}$ decay mode makes it especially interesting from an experimental point of view.

### Baryon-antibaryon enhancement near threshold

Many *B*-meson decays to baryonic final states present a characteristic enhancement at (production) threshold in the baryon-antibaryon mass spectrum of multi-body decays^4–7^, a fact that is still not fully understood. Such enhancements are not observed in mesonic final states. This same baryon-antibaryon enhancement near threshold is expected to be present in the decays of *b* baryons too. It awaits experimental confirmation.

**Figure 1:**
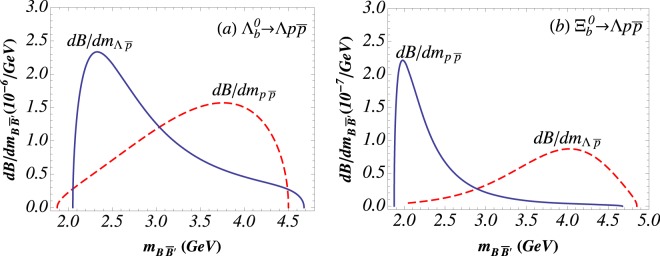
The dibaryon invariant mass spectra for the (**a**) ${{\rm{\Lambda }}}_{b}^{0}\to {\rm{\Lambda }}p\bar{p}$ and (**b**) ${{\Xi }}_{b}^{0}\to {\rm{\Lambda }}p\bar{p}$ decays.

Because of the participating Feynman diagrams, the ${{\rm{\Lambda }}}_{b}^{0}\to {\rm{\Lambda }}p\bar{p}$ and ${{\Xi }}_{b}^{0}\to {\rm{\Lambda }}p\bar{p}$ decay modes are expected not to exhibit a threshold enhancement in the same baryon-antibaryon system. A threshold enhancement in ${\rm{\Lambda }}\bar{p}$ is expected for the ${{\rm{\Lambda }}}_{b}^{0}\to {\rm{\Lambda }}p\bar{p}$ decay whereas it is the invariant mass of the $p\bar{p}$ system that is expected to peak near threshold in the case of the ${{\Xi }}_{b}^{0}\to {\rm{\Lambda }}p\bar{p}$ decay. The expected dibaryon invariant mass spectra are displayed in Fig. 1. These are clear signatures of the underlying QCD phenomenological framework used.

## Discussion

The study of hadronic decays of *b* hadrons has proved to be a rich playground for a better understanding of *CP* violation and for searches of manifestations of physics beyond the Standard Model. The study of charmless decays, in particular, has provided a wealth of crucial results and milestones in flavour physics, notably the discovery of direct *CP* violation in the ${B}^{0}\to {K}^{+}{\pi }^{-}$ decay^8,9^, the first observation of *CP* violation in the ${B}_{s}^{0}$-meson system^10^, and first evidence for *CP* violation in the decay of a baryon, *i*.*e*., in the decay of a spin-carrying particle^1^. Charmless decays, namely decays to final states with no charm flavour content, typically involve flavour charged ($b\to u$) and neutral ($b\to s$ and $b\to d$) transitions, which are suppressed with respect to the favoured $b\to c$ transition to open-charm final states.

In the years to come, the LHCb collaboration is the only running experiment capable of studying beauty baryons. We urge the collaboration to expand its presently ongoing programme of studies of *b*-hadron decays and to investigate purely baryonic decays, which, for the first time, would allow a study of *CP* violation in decay processes involving only spin-carrying particles. These yet unexplored elementary processes may hold key information towards a better understanding of the *CP* violating phenomena that are needed in order to explain the observed matter and antimatter asymmetry of the Universe.

The decay modes ${{\rm{\Lambda }}}_{b}^{0}\to {\rm{\Lambda }}p\bar{p}$ and ${{\Xi }}_{b}^{0}\to {\rm{\Lambda }}p\bar{p}$ are the most promising candidates for the first observation of decay processes exclusively involving spin-carrying particles. For what concerns *CP* violation, although the current sensitivity of the LHCb experiment is unlikely to reach the level predicted in the Standard Model, it is still worthy to explore the *CP* violating asymmetries of fully reconstructed baryonic decays as they could be large in models of physics beyond the Standard Model.

## Methods

**Figure 2:**
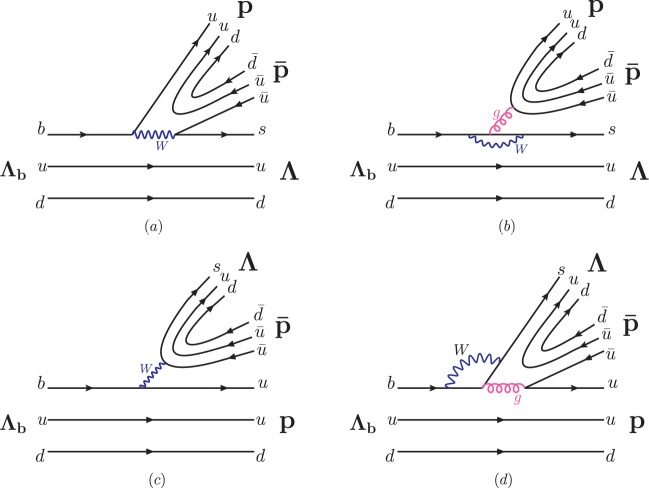
Feynman diagrams describing the purely baryonic decay ${{\rm{\Lambda }}}_{b}^{0}\to {\rm{\Lambda }}p\bar{p}$.

Figure 2 displays the dominant Feynman diagrams describing the purely baryonic decays ${{\bf{B}}}_{b}\to {{\bf{B}}}_{1}{\bar{{\bf{B}}}}_{2}{{\bf{B}}}_{3}$ (**B** denotes a baryon), which proceed with a ${{\bf{B}}}_{{\bf{b}}}\to {{\bf{B}}}_{{\bf{3}}}$ transition and a ${{\bf{B}}}_{{\bf{1}}}{\bar{{\bf{B}}}}_{{\bf{2}}}$-pair production. The decay mode ${{\rm{\Lambda }}}_{b}^{0}\to {\rm{\Lambda }}p\bar{p}$ is taken as an example. Similar diagrams can be drawn for the ${{\Xi }}_{b}^{0}\to {\rm{\Lambda }}p\bar{p}$ decay.

According to Fig. 2, the typical amplitude combines two matrix elements: $${\mathscr{A}}({{\bf{B}}}_{{\bf{b}}}\to {{\bf{B}}}_{{\bf{1}}}{\bar{{\bf{B}}}}_{{\bf{2}}}{{\bf{B}}}_{{\bf{3}}})\sim \langle {{\bf{B}}}_{{\bf{1}}}{\bar{{\bf{B}}}}_{{\bf{2}}}|({\bar{q}}_{1}{q}_{2})|0\rangle $$$$\langle {{\bf{B}}}_{{\bf{3}}}|({\bar{q}}_{3}b)|{{\bf{B}}}_{{\bf{b}}}\rangle $$, where $$({\bar{q}}_{1}{q}_{2})\,({\bar{q}}_{3}b)$$ are (axial)vector or (pseudo)scalar currents from the quark-level effective Hamiltonian for charmless $$b\to {q}_{1}{\bar{q}}_{2}{q}_{3}$$ transitions. In the amplitude, the two matrix elements can be further presented as the timelike baryonic form factors and the $${{\bf{B}}}_{{\bf{b}}}\to {{\bf{B}}}_{{\bf{3}}}$$ transition form factors^11–13^, together with the parameter for factorisable effects, being decomposed as effective Wilson coefficients^14^, the Fermi constant and Cabibbo-Kobayashi-Maskawa (CKM) matrix elements^15,16^. The extractions of the form factors with their uncertainties can be found in refs^11–13^. The form factors have been used to calculate $$ {\mathcal B} ({\bar{B}}_{s}^{0}\to {\rm{\Lambda }}\bar{p}{K}^{+},\bar{{\rm{\Lambda }}}p{K}^{-})$$^11^, whose value is in agreement with the measurement published by the LHCb collaboration^6^. Likewise, the prediction of the branching fraction $$ {\mathcal B} ({\bar{B}}^{0}\to {\rm{\Lambda }}\bar{p}{K}^{+}{K}^{-})$$ has been validated by the recently measurement by the Belle collaboration^17^.

Following the techniques described in previous work^3^, the branching fractions for the three-body purely baryonic decays discussed in this letter are predicted to be in the range 10^−7^–10^−6^, specifically $$ {\mathcal B} ({{\rm{\Lambda }}}_{b}^{0}\to {\rm{\Lambda }}p\bar{p})=$$$$({3.2}_{-0.3}^{+0.8}\pm 0.4\pm 0.7)\times {10}^{-6}$$ and $$ {\mathcal B} ({{\Xi }}_{b}^{0}\to {\rm{\Lambda }}p\bar{p})=(1.4\pm 0.1\pm 0.1\pm 0.4)\times {10}^{-7}$$, where the first uncertainties account for non-factorizable effects, the second reflect the experimental knowledge of the CKM matrix elements, and the third arise from those on the form factors^11–13^.

The direct *CP* violating rate (Γ) asymmetry can be defined by

1 $${{\mathscr{A}}}_{CP}=\frac{{\rm{\Gamma }}({{\bf{B}}}_{h}\to {{\bf{B}}}_{{l}_{1}}{\bar{{\bf{B}}}}_{{l}_{2}}{{\bf{B}}}_{{l}_{3}})-{\rm{\Gamma }}({\bar{{\bf{B}}}}_{h}\to {\bar{{\bf{B}}}}_{{l}_{1}}{{\bf{B}}}_{{l}_{2}}{\bar{{\bf{B}}}}_{{l}_{3}})}{{\rm{\Gamma }}({{\bf{B}}}_{h}\to {{\bf{B}}}_{{l}_{1}}{\bar{{\bf{B}}}}_{{l}_{2}}{{\bf{B}}}_{{l}_{3}})+{\rm{\Gamma }}({\bar{{\bf{B}}}}_{h}\to {\bar{{\bf{B}}}}_{{l}_{1}}{{\bf{B}}}_{{l}_{2}}{\bar{{\bf{B}}}}_{{l}_{3}})}.$$

If both weak (*γ*) and strong (*δ*) phases are non-vanishing, one has that ${{\mathscr{A}}}_{CP}\propto \,\sin \,\gamma \,\sin \,\delta$. The direct *CP* asymmetries of ${{\rm{\Lambda }}}_{b}^{0}\to {\rm{\Lambda }}p\bar{p}$ and ${{\Xi }}_{b}^{0}\to {\rm{\Lambda }}p\bar{p}$ decays are predicted to be $(3.4\pm 0.1\pm 0.1\pm 1.0) \%$ and $(\,-\,13.0\pm 0.5\pm 1.5\pm 1.1) \%$, respectively, with the uncertainties mentioned early.
